# 1,10,10-Trimethyl-5-phenyl-3-oxa-4-aza­tri­cyclo­[5.2.1.0^2,6^]dec-4-en-2-ol

**DOI:** 10.1107/S160053681302000X

**Published:** 2013-07-24

**Authors:** Brahim Boualy, Mohamed Anouar Harrad, Abdelghani Oudahmane, Ahmed Benharref, Moha Berraho

**Affiliations:** a Laboratoire de Chimie de Coordination, Faculté des Sciences-Semlalia, BP 2390, 40001 Marrakech, Morocco; b Laboratoire des Matériaux Inorganiques, UMR CNRS 6002, Université Blaise Pascal, 24 Avenue des Landais, 63177 Aubière, France; c Laboratoire de Chimie des Substances Naturelles, Unité Associé au CNRST (URAC16), Faculté des Sciences-Semlalia, BP 2390, Boulevard My Abdellah, 40000 Marrakech, Morocco

## Abstract

The title compound, C_17_H_21_NO_2_, was synthesized by the reaction of (1*R*)-(+)-3-benzyl­camphor and hydroxyl­amine. The oxazole ring makes a dihedral angle of 23.42 (16)° with the phenyl ring. The six-membered ring of the norboryl group adopts a boat conformation, whereas each of the five-membered rings of the norboryl group displays a flattened envelope conformation, with the C atom carrying the methyl groups representing the flap for both rings. In the crystal, mol­ecules are linked into zigzag chains propagating along the *b* axis by O—H⋯N hydrogen bonds.

## Related literature

For the functionalization of camphor, see: Jennings & Herschbach (1965 ▶); Pastrán *et al.*, (2011 ▶). For transition metal complexes of camphor, see: Spannenberg *et al.* (2002 ▶); Harrad *et al.* (2010 ▶); Ait Ali *et al.* (2006 ▶); Gaudo *et al.* (2011 ▶). For ring-puckering parameters, see: Cremer & Pople (1975 ▶).
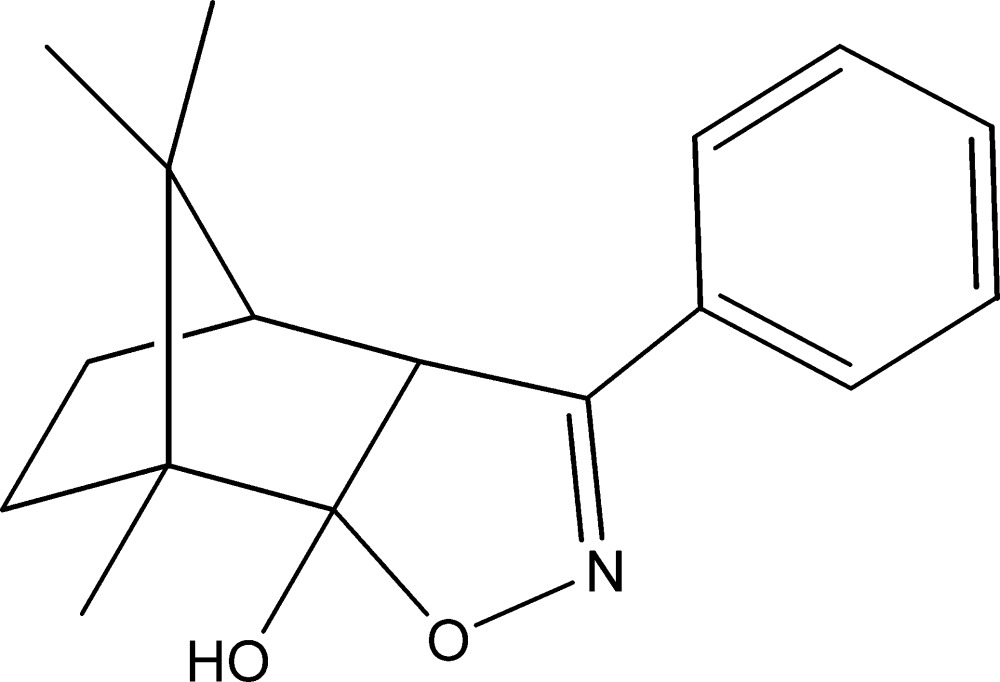


## Experimental

### 

#### Crystal data

C_17_H_21_NO_2_
*M* *_r_* = 271.35
Monoclinic, 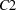

*a* = 22.1681 (18) Å
*b* = 6.6134 (5) Å
*c* = 10.7358 (8) Å
β = 108.277 (3)°
*V* = 1494.5 (2) Å^3^
*Z* = 4
Mo *K*α radiation
μ = 0.08 mm^−1^
*T* = 296 K
0.58 × 0.34 × 0.14 mm

#### Data collection

Bruker APEXII CCD diffractometer
Absorption correction: multi-scan (*SADABS*; Sheldrick, 2008 ▶) *T*_min_ = 0.627, *T*_max_ = 0.745
4379 measured reflections
1350 independent reflections
1220 reflections with *I* > 2σ(*I*)
*R*_int_ = 0.025

#### Refinement

*R*[*F* ^2^ > 2σ(*F* ^2^)] = 0.042
*wR*(*F* ^2^) = 0.106
*S* = 1.08
1350 reflections
186 parameters
1 restraint
H-atom parameters constrained
Δρ_max_ = 0.29 e Å^−3^
Δρ_min_ = −0.24 e Å^−3^

### 

Data collection: *APEX2* (Bruker, 2009 ▶); cell refinement: *SAINT* (Bruker, 2009 ▶); data reduction: *SAINT*; program(s) used to solve structure: *SHELXS97* (Sheldrick, 2008 ▶); program(s) used to refine structure: *SHELXL97* (Sheldrick, 2008 ▶); molecular graphics: *ORTEP-3 for Windows* (Farrugia, 2012 ▶)and *PLATON* (Spek, 2009 ▶); software used to prepare material for publication: *WinGX* (Farrugia, 2012 ▶).

## Supplementary Material

Crystal structure: contains datablock(s) I, global. DOI: 10.1107/S160053681302000X/bt6921sup1.cif

Additional supplementary materials:  crystallographic information; 3D view; checkCIF report

## Figures and Tables

**Table 1:** Hydrogen-bond geometry (Å, °)

*D*—H⋯*A*	*D*—H	H⋯*A*	*D*⋯*A*	*D*—H⋯*A*
O2—H2⋯N2^i^	0.82	2.06	2.877 (3)	174

Symmetry code: (i) 
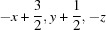
.

**Fig. 1.**
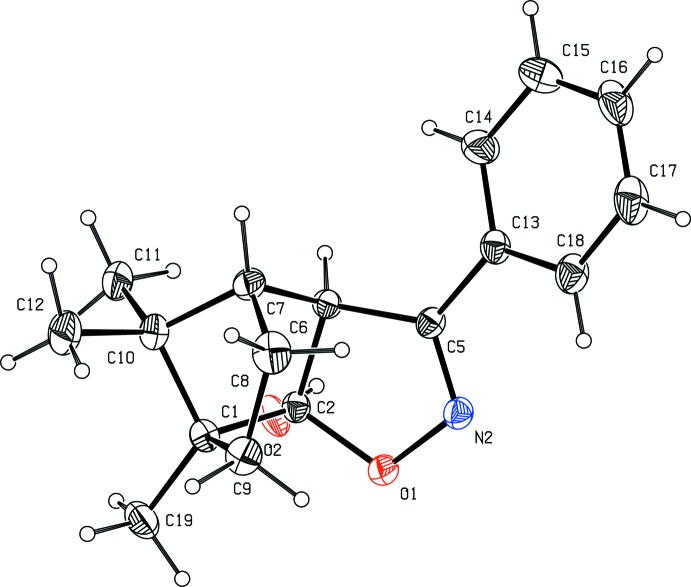
Molecular structure of the title compound with the atom-labelling scheme. Displacement ellipsoids are drawn at the 30% probability. level. H atoms are represented as small spheres of arbitrary radii.

**Fig. 2.**
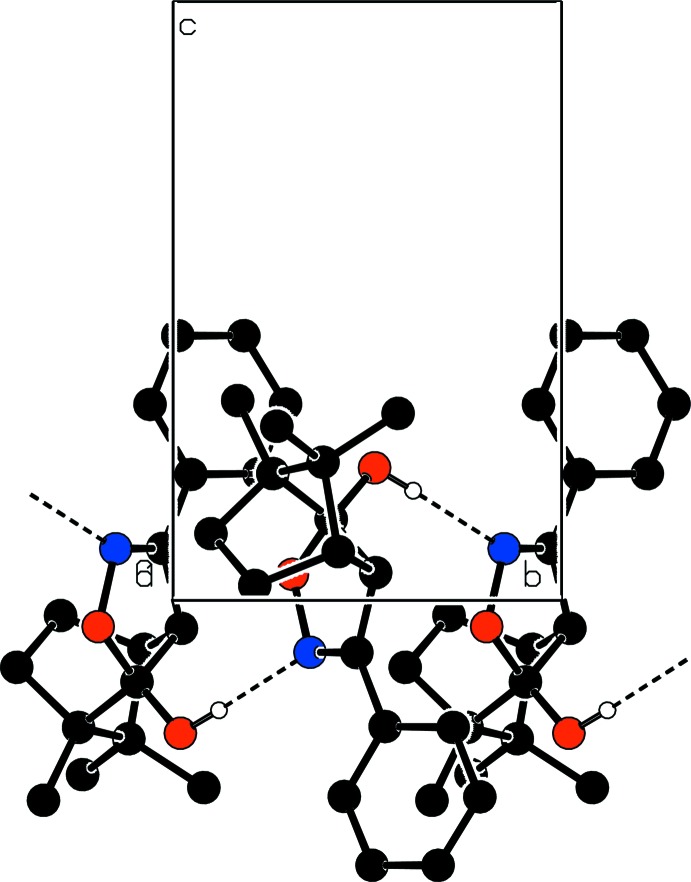
Partial packing view showing the O—H···N and N—H···N interactions (dashed lines) and the formation of a chain parallel to the b axis. H atoms not involved in hydrogen bonding have been omitted for clarity.

## supplementary crystallographic information

### Comment

The versatility and importance of camphor as a chiral starting material in the synthesis of natural products is primarily due to the availability of methods for the introduction of functional groups (Jennings & Herschbach, 1965; Pastrán *et al.*, 2011). We have developed a series of complexes based on camphor 1,3-diketonato ligands (Spannenberg *et al.*, 2002; Harrad *et al.*, 2010; Ait Ali *et al.*, 2006), and their application in catalytic asymmetric reactions has been described (Gaudo *et al.*, 2011). In this work, we present the structure of a new heterocyclic compound (1,10,10-trimethyl-5-phenyl-3-oxa-4-aza-tricyclo[5.2.1.02,6]dec-4- en-2-ol)) which we have synthesized by hetercyclization from benzylcamphor with hydroxylamine. In the molecule (Fig. 1), the six-membered ring of the norboryl system adopts a boat conformation, as indicated by Cremer & Pople (1975) puckering parameters Q = 0.966 (3) Å and spherical polar angle θ = 89.71 (17)° with φ = 121.07 (19)°. The two fused five-membered rings display an envelope conformation with Q = 0.602 (3) Å and φ = 287.7 (3)° for the first ring (C6, C7, C9, C10, C12) and Q = 0.590 (4) Å and φ = 37.7 (4)° for the other ring (C7, C10, C12, C14, C19). In the crystal structure, molecules are linked into zigzag chains (Fig. 2) running along the *b* axis by intermolecular O—H···N hydrogen bonds (Table 1).

### Experimental

(1*R*)-(+)-3-benzyl-camphor (1 mmol), and hydroxylamine (2 mmol), in dichloromethane (10 ml) were vigorously stirred at reflux. The progress of the reaction was followed by TLC. The reaction went to completion after 24 h. After completion of the reaction, the mixture was diluted with H_2_O (10 ml) and extracted with EtOAc (2 × 10 ml) and dried over Na_2_SO_4_. The title compound was isolated as a white powder by column chromatography on silica gel using ethyl acetate–n-hexane as eluant (yield 79%; m.p. = 145°C). Colourless single crystals suitable for X-ray analysis were obtained by slow evaporation of n-hexane solution.

### Refinement

All H atoms were fixed geometrically and treated as riding with O—H = 0.82 Å, C—H = 0.96 Å (methyl), 0.97 Å (methylene), 0.98 Å (methine) with *U*_iso_(H) = 1.2*U*_eq_(methylene, methine) or *U*_iso_(H) = 1.5*U*_eq_(methyl). The torsion angle about the C—O bond of the hydroxyl group was refined. In the absence of significant anomalous scattering, the absolute configuration could not be reliably determined and thus Friedel pairs were merged and any references to the Flack parameter were removed.

### Crystal data

**Table:**

C_17_H_21_NO_2_	*F*(000) = 584
*M**_r_* = 271.35	*D*_x_ = 1.202 Mg m^−^^3^
Monoclinic, *C*2	Mo *K*α radiation, λ = 0.71073 Å
Hall symbol: C 2y	Cell parameters from 2921 reflections
*a* = 22.1681 (18) Å	θ = 3.2–24.5°
*b* = 6.6134 (5) Å	µ = 0.08 mm^−^^1^
*c* = 10.7358 (8) Å	*T* = 296 K
β = 108.277 (3)°	Plaquet, colourless
*V* = 1494.5 (2) Å^3^	0.58 × 0.34 × 0.14 mm
*Z* = 4	

### Data collection

**Table:**

Bruker APEXII CCD diffractometer	1350 independent reflections
Radiation source: fine-focus sealed tube	1220 reflections with *I* > 2σ(*I*)
Graphite monochromator	*R*_int_ = 0.025
Detector resolution: 8.3333 pixels mm^-1^	θ_max_ = 24.5°, θ_min_ = 3.2°
ω and φ scans	*h* = −25→25
Absorption correction: multi-scan (*SADABS*; Sheldrick, 2008)	*k* = −6→7
*T*_min_ = 0.627, *T*_max_ = 0.745	*l* = −12→12
4379 measured reflections	

### Refinement

**Table:**

Refinement on *F*^2^	Secondary atom site location: difference Fourier map
Least-squares matrix: full	Hydrogen site location: inferred from neighbouring sites
*R*[*F*^2^ > 2σ(*F*^2^)] = 0.042	H-atom parameters constrained
*wR*(*F*^2^) = 0.106	*w* = 1/[σ^2^(*F*_o_^2^) + (0.0701*P*)^2^ + 0.2932*P*] where *P* = (*F*_o_^2^ + 2*F*_c_^2^)/3
*S* = 1.08	(Δ/σ)_max_ < 0.001
1350 reflections	Δρ_max_ = 0.29 e Å^−^^3^
186 parameters	Δρ_min_ = −0.24 e Å^−^^3^
1 restraint	Extinction correction: *SHELXL97* (Sheldrick, 2008), Fc^*^=kFc[1+0.001xFc^2^λ^3^/sin(2θ)]^-1/4^
Primary atom site location: structure-invariant direct methods	Extinction coefficient: 0.050 (5)

### Special details

**Table:**

Geometry. All s.u.'s (except the s.u. in the dihedral angle between two l.s. planes) are estimated using the full covariance matrix. The cell s.u.'s are taken into account individually in the estimation of s.u.'s in distances, angles and torsion angles; correlations between s.u.'s in cell parameters are only used when they are defined by crystal symmetry. An approximate (isotropic) treatment of cell s.u.'s is used for estimating s.u.'s involving l.s. planes.
Refinement. Refinement of *F*^2^ against ALL reflections. The weighted *R*-factor *wR* and goodness of fit *S* are based on *F*^2^, conventional *R*-factors *R* are based on *F*, with *F* set to zero for negative *F*^2^. The threshold expression of *F*^2^ > 2σ(*F*^2^) is used only for calculating *R*-factors(gt) *etc*. and is not relevant to the choice of reflections for refinement. *R*-factors based on *F*^2^ are statistically about twice as large as those based on *F*, and *R*-factors based on ALL data will be even larger.

### Fractional atomic coordinates and isotropic or equivalent isotropic displacement parameters (Å^2^)

**Table:**

	*x*	*y*	*z*	*U*_iso_*/*U*_eq_	
C5	0.81485 (10)	0.9730 (4)	−0.0870 (2)	0.0383 (6)	
C6	0.85865 (11)	1.0215 (4)	0.0464 (2)	0.0393 (6)	
H6	0.8609	1.1673	0.0637	0.047*	
C7	0.92540 (11)	0.9251 (5)	0.0815 (2)	0.0527 (8)	
H7	0.9569	1.0084	0.0585	0.063*	
C8	0.91859 (14)	0.7103 (6)	0.0251 (3)	0.0632 (9)	
H8A	0.8924	0.7088	−0.0662	0.076*	
H8B	0.9597	0.6520	0.0325	0.076*	
C9	0.88635 (13)	0.5975 (5)	0.1116 (3)	0.0560 (7)	
H9A	0.9122	0.4844	0.1557	0.067*	
H9B	0.8450	0.5473	0.0598	0.067*	
C1	0.87986 (11)	0.7567 (4)	0.2119 (2)	0.0445 (7)	
C2	0.82786 (10)	0.9070 (4)	0.1358 (2)	0.0387 (6)	
C10	0.94087 (11)	0.8860 (5)	0.2308 (2)	0.0520 (7)	
C12	1.00340 (13)	0.7670 (7)	0.2908 (3)	0.0777 (11)	
H12A	1.0381	0.8412	0.2775	0.116*	
H12B	1.0001	0.6374	0.2490	0.116*	
H12C	1.0107	0.7487	0.3831	0.116*	
C11	0.94630 (15)	1.0792 (6)	0.3124 (3)	0.0690 (9)	
H11A	0.9852	1.1475	0.3175	0.104*	
H11B	0.9461	1.0448	0.3991	0.104*	
H11C	0.9110	1.1664	0.2716	0.104*	
C13	0.82295 (11)	1.0459 (5)	−0.2104 (2)	0.0443 (6)	
C18	0.79751 (13)	0.9407 (6)	−0.3273 (2)	0.0586 (8)	
H18	0.7746	0.8225	−0.3287	0.070*	
C17	0.80638 (16)	1.0122 (7)	−0.4421 (3)	0.0738 (12)	
H17	0.7889	0.9415	−0.5201	0.089*	
C16	0.83984 (16)	1.1823 (8)	−0.4422 (3)	0.0794 (12)	
H16	0.8461	1.2269	−0.5193	0.095*	
C15	0.86471 (18)	1.2897 (8)	−0.3279 (4)	0.0852 (12)	
H15	0.8870	1.4086	−0.3283	0.102*	
C14	0.85657 (16)	1.2210 (6)	−0.2119 (3)	0.0671 (9)	
H14	0.8739	1.2935	−0.1347	0.080*	
C19	0.86922 (15)	0.6664 (6)	0.3337 (3)	0.0625 (9)	
H19A	0.8294	0.5958	0.3092	0.094*	
H19B	0.8686	0.7726	0.3942	0.094*	
H19C	0.9030	0.5738	0.3745	0.094*	
O1	0.77279 (7)	0.8072 (3)	0.04364 (16)	0.0467 (5)	
N2	0.77018 (9)	0.8524 (4)	−0.08639 (18)	0.0438 (6)	
O2	0.80582 (8)	1.0188 (3)	0.22155 (14)	0.0519 (6)	
H2	0.7864	1.1178	0.1833	0.078*	

### Atomic displacement parameters (Å^2^)

**Table:**

	*U*^11^	*U*^22^	*U*^33^	*U*^12^	*U*^13^	*U*^23^
C5	0.0383 (11)	0.0409 (14)	0.0357 (11)	−0.0014 (11)	0.0116 (9)	−0.0065 (11)
C6	0.0455 (12)	0.0374 (14)	0.0336 (11)	−0.0048 (11)	0.0103 (9)	−0.0014 (11)
C7	0.0380 (12)	0.070 (2)	0.0502 (14)	−0.0076 (14)	0.0144 (10)	0.0066 (15)
C8	0.0559 (15)	0.075 (2)	0.0647 (17)	0.0157 (17)	0.0276 (13)	−0.0043 (17)
C9	0.0555 (14)	0.0465 (17)	0.0643 (17)	0.0097 (14)	0.0165 (12)	−0.0061 (15)
C1	0.0451 (12)	0.0443 (17)	0.0422 (13)	0.0041 (12)	0.0109 (10)	0.0047 (12)
C2	0.0386 (11)	0.0430 (16)	0.0341 (11)	0.0009 (11)	0.0107 (9)	−0.0011 (11)
C10	0.0428 (12)	0.059 (2)	0.0478 (13)	0.0021 (14)	0.0047 (10)	0.0047 (15)
C12	0.0453 (14)	0.098 (3)	0.077 (2)	0.0091 (18)	0.0011 (13)	0.015 (2)
C11	0.0690 (17)	0.068 (2)	0.0520 (16)	−0.0134 (19)	−0.0069 (12)	0.0013 (17)
C13	0.0453 (12)	0.0521 (17)	0.0358 (12)	0.0018 (13)	0.0130 (9)	−0.0030 (12)
C18	0.0577 (14)	0.078 (2)	0.0395 (13)	−0.0028 (16)	0.0148 (11)	−0.0094 (15)
C17	0.0720 (18)	0.116 (4)	0.0356 (14)	0.009 (2)	0.0197 (13)	−0.0077 (18)
C16	0.0704 (19)	0.126 (4)	0.0463 (17)	0.013 (2)	0.0247 (14)	0.024 (2)
C15	0.090 (2)	0.097 (3)	0.075 (2)	−0.017 (2)	0.0348 (18)	0.024 (2)
C14	0.0841 (19)	0.070 (2)	0.0478 (15)	−0.019 (2)	0.0218 (13)	0.0020 (16)
C19	0.0637 (16)	0.066 (2)	0.0557 (16)	0.0106 (17)	0.0162 (13)	0.0225 (17)
O1	0.0397 (8)	0.0571 (13)	0.0423 (9)	−0.0071 (9)	0.0115 (6)	0.0039 (8)
N2	0.0418 (10)	0.0513 (15)	0.0371 (10)	−0.0040 (11)	0.0107 (8)	−0.0025 (10)
O2	0.0621 (11)	0.0616 (14)	0.0337 (9)	0.0215 (10)	0.0174 (7)	0.0054 (9)

### Geometric parameters (Å, º)

**Table:**

C5—N2	1.273 (3)	C12—H12A	0.9600
C5—C13	1.473 (3)	C12—H12B	0.9600
C5—C6	1.492 (3)	C12—H12C	0.9600
C6—C2	1.540 (3)	C11—H11A	0.9600
C6—C7	1.545 (4)	C11—H11B	0.9600
C6—H6	0.9800	C11—H11C	0.9600
C7—C8	1.534 (5)	C13—C14	1.380 (5)
C7—C10	1.552 (4)	C13—C18	1.390 (4)
C7—H7	0.9800	C18—C17	1.391 (4)
C8—C9	1.532 (4)	C18—H18	0.9300
C8—H8A	0.9700	C17—C16	1.348 (6)
C8—H8B	0.9700	C17—H17	0.9300
C9—C1	1.545 (4)	C16—C15	1.374 (6)
C9—H9A	0.9700	C16—H16	0.9300
C9—H9B	0.9700	C15—C14	1.390 (4)
C1—C19	1.522 (4)	C15—H15	0.9300
C1—C2	1.547 (3)	C14—H14	0.9300
C1—C10	1.558 (4)	C19—H19A	0.9600
C2—O2	1.384 (3)	C19—H19B	0.9600
C2—O1	1.465 (3)	C19—H19C	0.9600
C10—C11	1.532 (5)	O1—N2	1.411 (3)
C10—C12	1.548 (4)	O2—H2	0.8200
			
N2—C5—C13	121.6 (2)	C11—C10—C1	116.2 (2)
N2—C5—C6	113.7 (2)	C12—C10—C1	113.8 (3)
C13—C5—C6	124.6 (2)	C7—C10—C1	93.18 (19)
C5—C6—C2	102.15 (19)	C10—C12—H12A	109.5
C5—C6—C7	115.5 (2)	C10—C12—H12B	109.5
C2—C6—C7	102.88 (19)	H12A—C12—H12B	109.5
C5—C6—H6	111.8	C10—C12—H12C	109.5
C2—C6—H6	111.8	H12A—C12—H12C	109.5
C7—C6—H6	111.8	H12B—C12—H12C	109.5
C8—C7—C6	108.5 (2)	C10—C11—H11A	109.5
C8—C7—C10	102.5 (3)	C10—C11—H11B	109.5
C6—C7—C10	101.8 (2)	H11A—C11—H11B	109.5
C8—C7—H7	114.2	C10—C11—H11C	109.5
C6—C7—H7	114.2	H11A—C11—H11C	109.5
C10—C7—H7	114.2	H11B—C11—H11C	109.5
C9—C8—C7	102.6 (2)	C14—C13—C18	118.5 (3)
C9—C8—H8A	111.2	C14—C13—C5	120.2 (2)
C7—C8—H8A	111.2	C18—C13—C5	121.3 (3)
C9—C8—H8B	111.2	C17—C18—C13	120.0 (3)
C7—C8—H8B	111.2	C17—C18—H18	120.0
H8A—C8—H8B	109.2	C13—C18—H18	120.0
C8—C9—C1	104.7 (3)	C16—C17—C18	121.0 (3)
C8—C9—H9A	110.8	C16—C17—H17	119.5
C1—C9—H9A	110.8	C18—C17—H17	119.5
C8—C9—H9B	110.8	C17—C16—C15	119.9 (3)
C1—C9—H9B	110.8	C17—C16—H16	120.1
H9A—C9—H9B	108.9	C15—C16—H16	120.1
C19—C1—C9	113.9 (3)	C16—C15—C14	120.2 (4)
C19—C1—C2	114.5 (2)	C16—C15—H15	119.9
C9—C1—C2	106.7 (2)	C14—C15—H15	119.9
C19—C1—C10	117.8 (2)	C13—C14—C15	120.4 (3)
C9—C1—C10	101.4 (2)	C13—C14—H14	119.8
C2—C1—C10	100.9 (2)	C15—C14—H14	119.8
O2—C2—O1	107.27 (17)	C1—C19—H19A	109.5
O2—C2—C6	118.0 (2)	C1—C19—H19B	109.5
O1—C2—C6	103.82 (17)	H19A—C19—H19B	109.5
O2—C2—C1	110.61 (19)	C1—C19—H19C	109.5
O1—C2—C1	113.1 (2)	H19A—C19—H19C	109.5
C6—C2—C1	104.03 (18)	H19B—C19—H19C	109.5
C11—C10—C12	106.6 (3)	N2—O1—C2	109.86 (17)
C11—C10—C7	113.8 (3)	C5—N2—O1	110.33 (18)
C12—C10—C7	113.2 (2)	C2—O2—H2	109.5

### Hydrogen-bond geometry (Å, º)

**Table:**

*D*—H···*A*	*D*—H	H···*A*	*D*···*A*	*D*—H···*A*
O2—H2···N2^i^	0.82	2.06	2.877 (3)	174

Symmetry code: (i) −*x*+3/2, *y*+1/2, −*z*.
